# Supplementary material for: Ubiquitin ligation to F-box protein targets by SCF–RBR E3–E3 super-assembly
Source: Nature. 2021 Feb 3;590(7847):671–6. doi: 10.1038/s41586-021-03197-9 (PMC7904520; doi:10.1038/s41586-021-03197-9)
Supplement: Supplementary file 1 — This file contains Supplementary Figures 1-3 and Supplementary Table 1. [file 41586_2021_3197_MOESM1_ESM.pdf]

nature portfolio

https://doi.org/10.1038/s41586-021-03197-9

Supplementary information

Ubiquitin ligation to F-box protein targets by SCF–RBR E3–E3 super-assembly

In the format provided by the authors and unedited

Supplementary Information

uncropped SDS PAGE gels

Supplementary Information Figure 1

Extended data figure 1b left

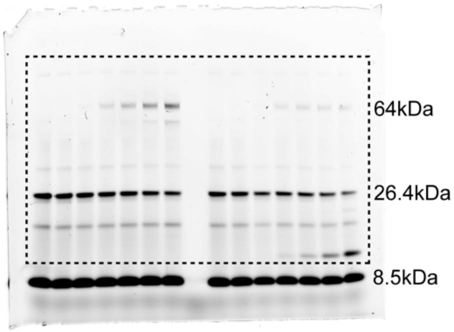

Extended data figure 1b right

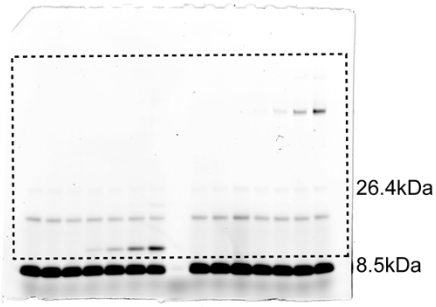

Extended data figure 1e

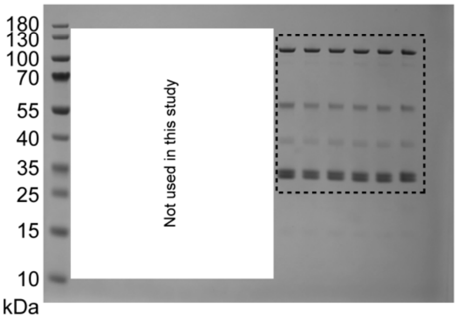

Extended data figure 1 f

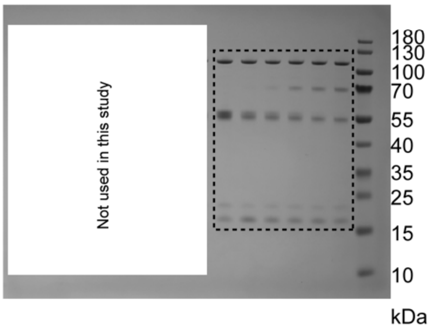

Extended data figure 2c

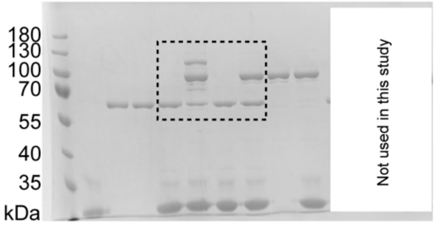

Extended data figure 2d

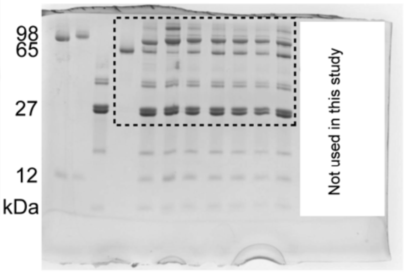

Extended data figure 2e

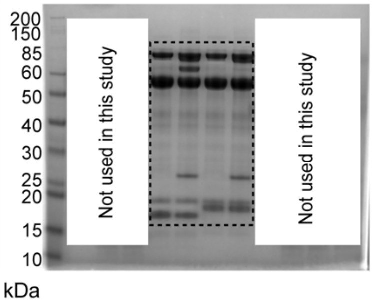

Extended data figure 2g

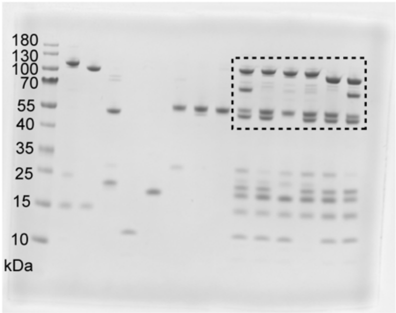

Extended data figure 2h

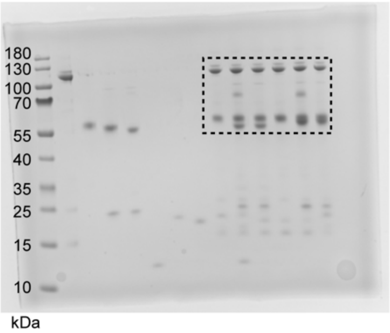

uncropped SDS PAGE gels

Supplementary Information Figure 1

Extended data figure 6b

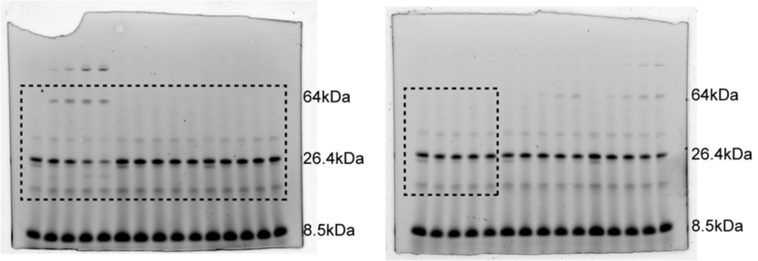

Extended data figure 6c left

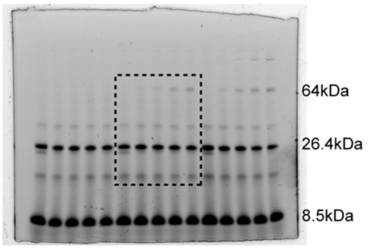

Extended data figure 6c middle & right

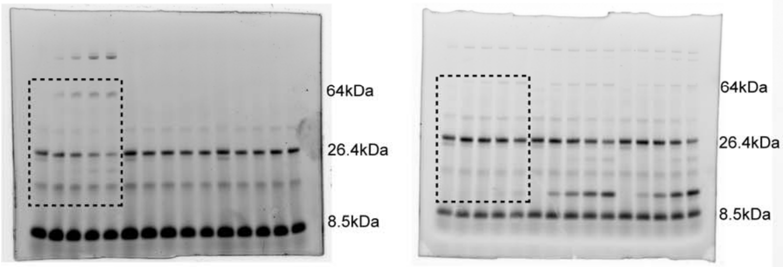

Extended data figure 6d left

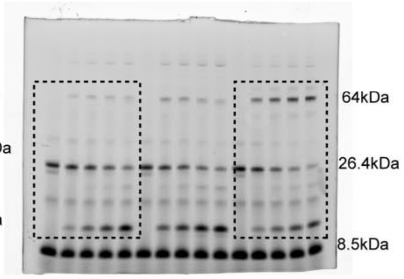

Extended data figure 6d right

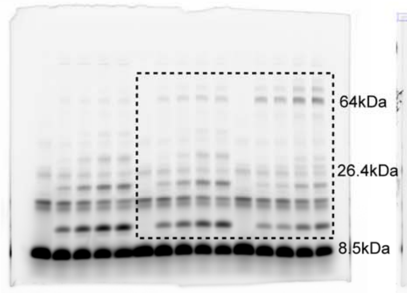

Extended data figure 6e top

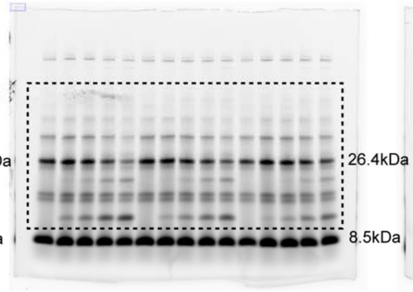

Extended data figure 6e bottom

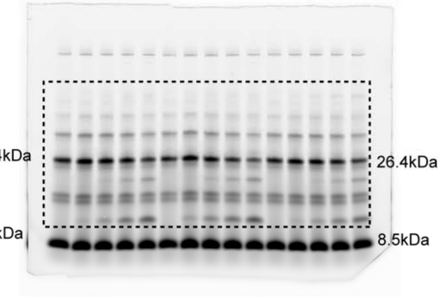

Extended data figure 6f

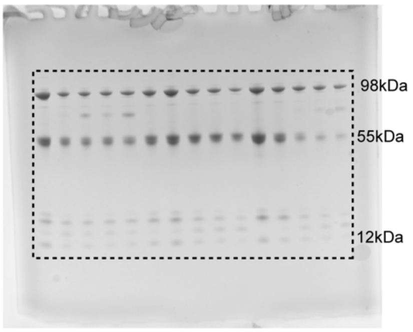

Extended data figure 7f

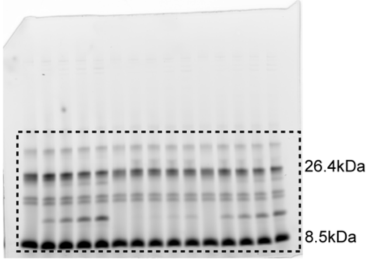

Extended data figure 7g

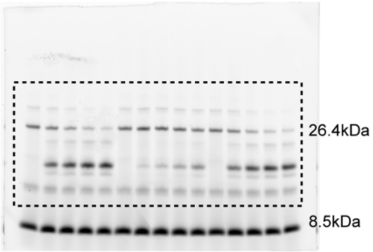

uncropped SDS PAGE gels

Supplementary Information Figure 1

Extended data figure 8a

ARIH1- CYCLIN E

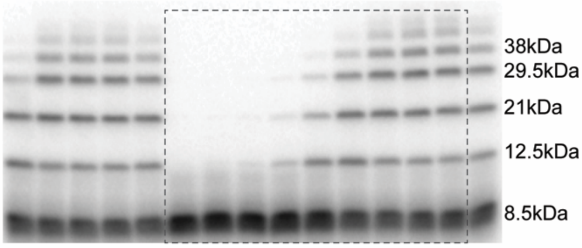

UBE2D-CYCLIN E

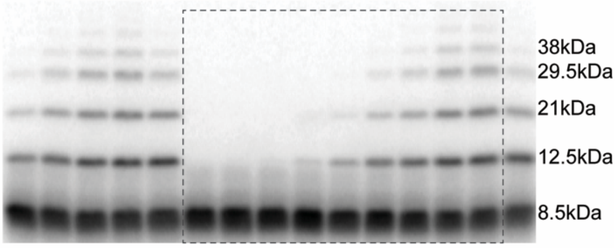

ARIH1- CYCLIN E UB

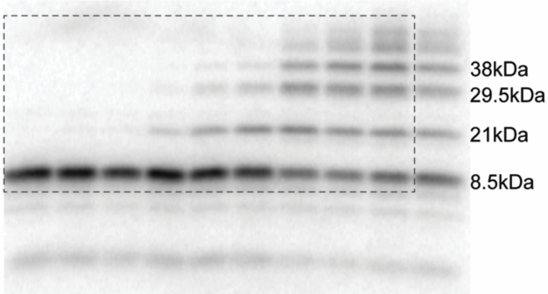

UBE2D-CYCLIN E UB

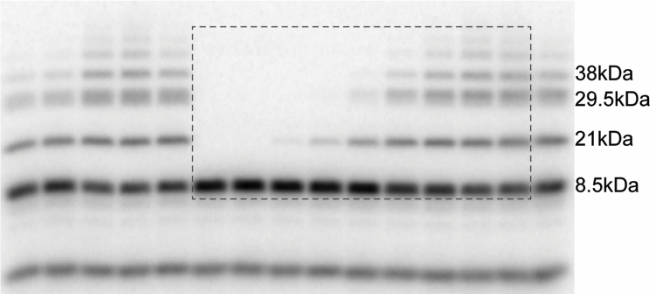

ARIH1- CYCLIN E short

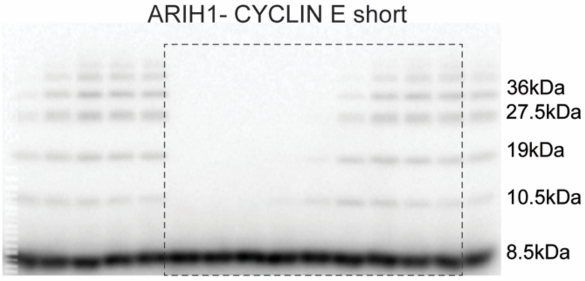

ARIH1- βCATENIN

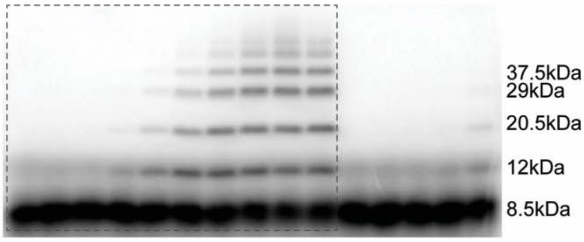

UBE2D- βCATENIN

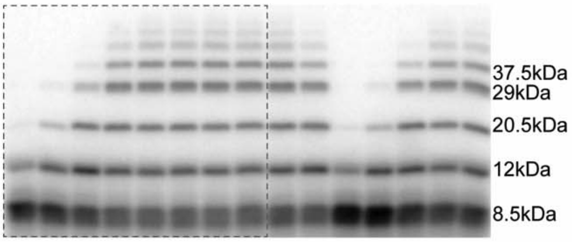

ARIH1- βCATENIN short

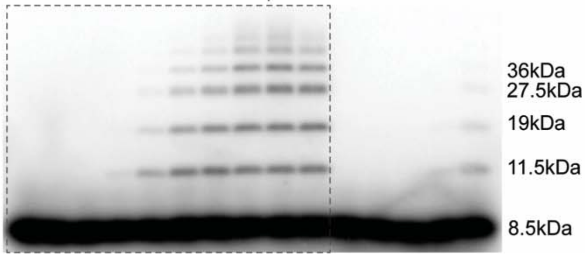

ARIH1- CRY1

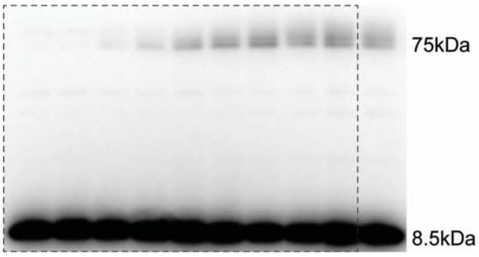

uncropped SDS PAGE gels

Supplementary Information Figure 1

Extended data figure 8b

ARIH1- CYCLIN E

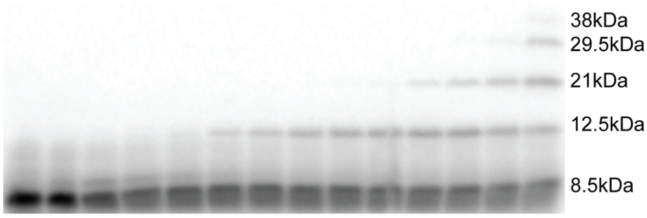

UBE2D-CYCLIN E

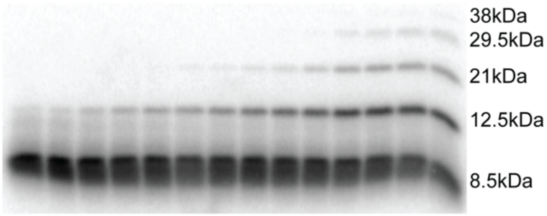

ARIH1- CYCLIN E UB

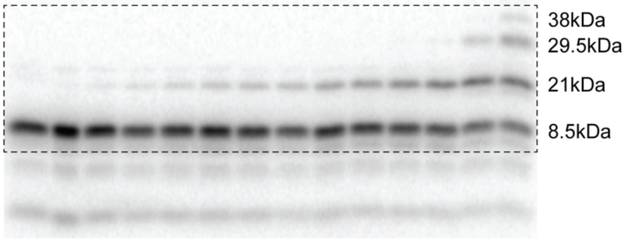

UBE2D-CYCLIN E UB

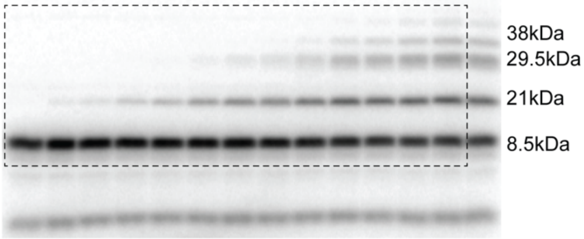

ARIH1- CYCLIN E short

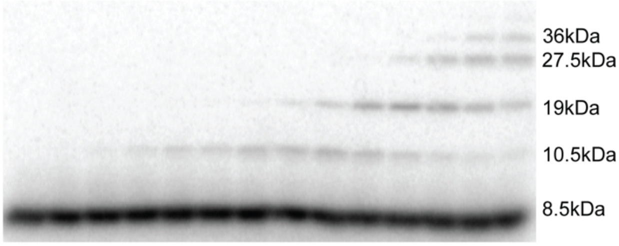

ARIH1- βCATENIN

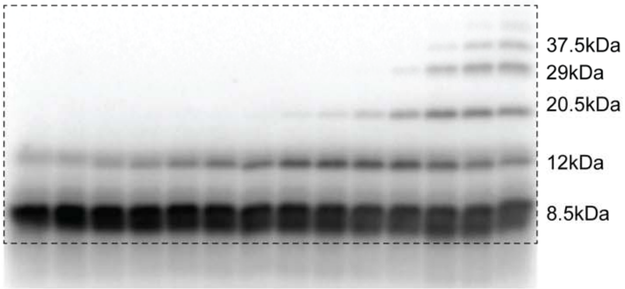

UBE2D- βCATENIN

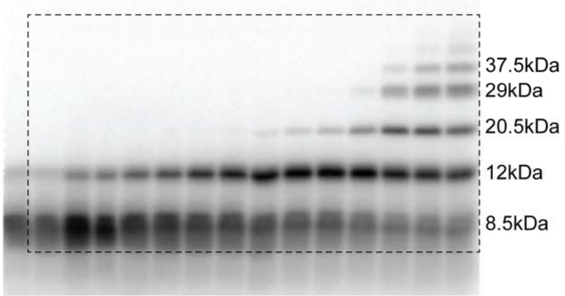

ARIH1- βCATENIN short

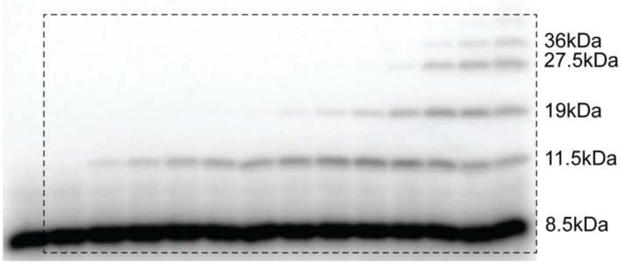

ARIH1- CRY1

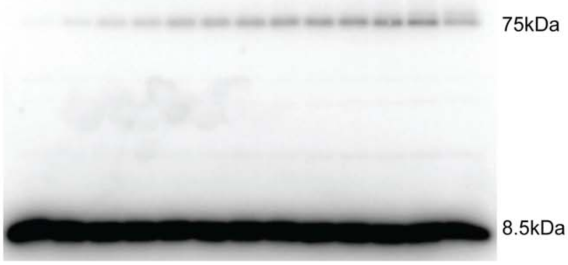

Transition State 1:

TS1 ABP - SCF^{SKP2}, Cyclin A, CDK2

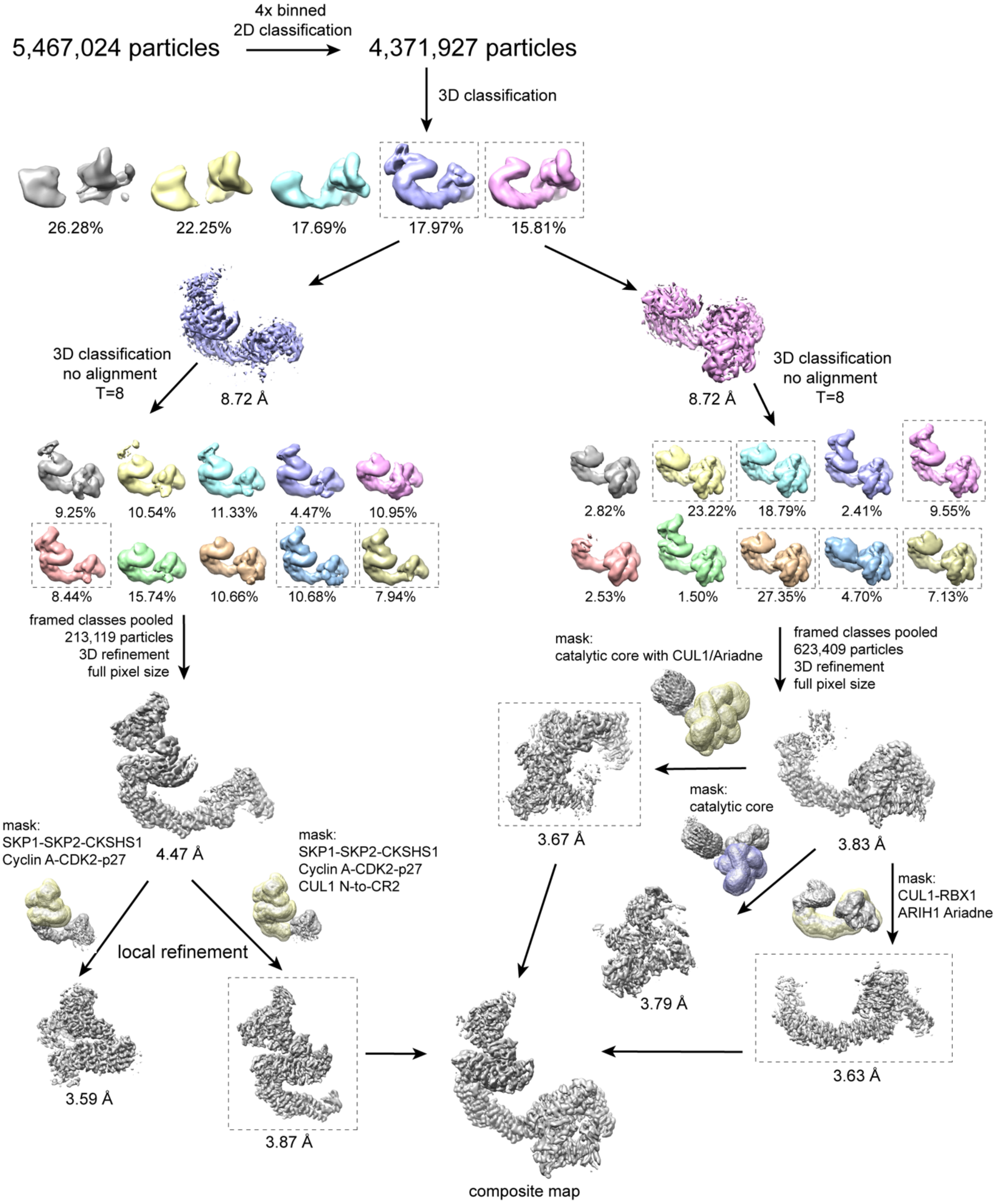

Supplemental Figure 2 | Cryo-EM image processing flowchart of the complex representing Transition State 1, neddylated SCF^{SKP2}-dependent ubiquitin transfer from the E2 UBE2L3 to ARIH1. **a,** Cryo-EM image processing flowchart for complex representing Transition State 1: UBE2L3~ubiquitin~ARIH1-NEDD8–CUL1-RBX1-SKP1-SKP2-CKSHS1-p27-Cyclin A-CDK2. Two distinct classes, one displaying prominent density for the substrate binding region and one for the E3-E3 catalytic core, were identified during 3D classification. Further classifications without image alignment yielded a more homogenous set of particles after similar classes had been pooled. Overall maps were refined to 4.47 Å and 3.83 Å, respectively. Refinements with local angular searches and masks covering defined subcomplexes, resulted in improved map quality and resolution. Selected focused maps were joined via PHENIX to obtain a complete composite map, enabling docking of prior crystal structures.

Transition state 2:

TS2 p27 ABP SCF^{SKP2}

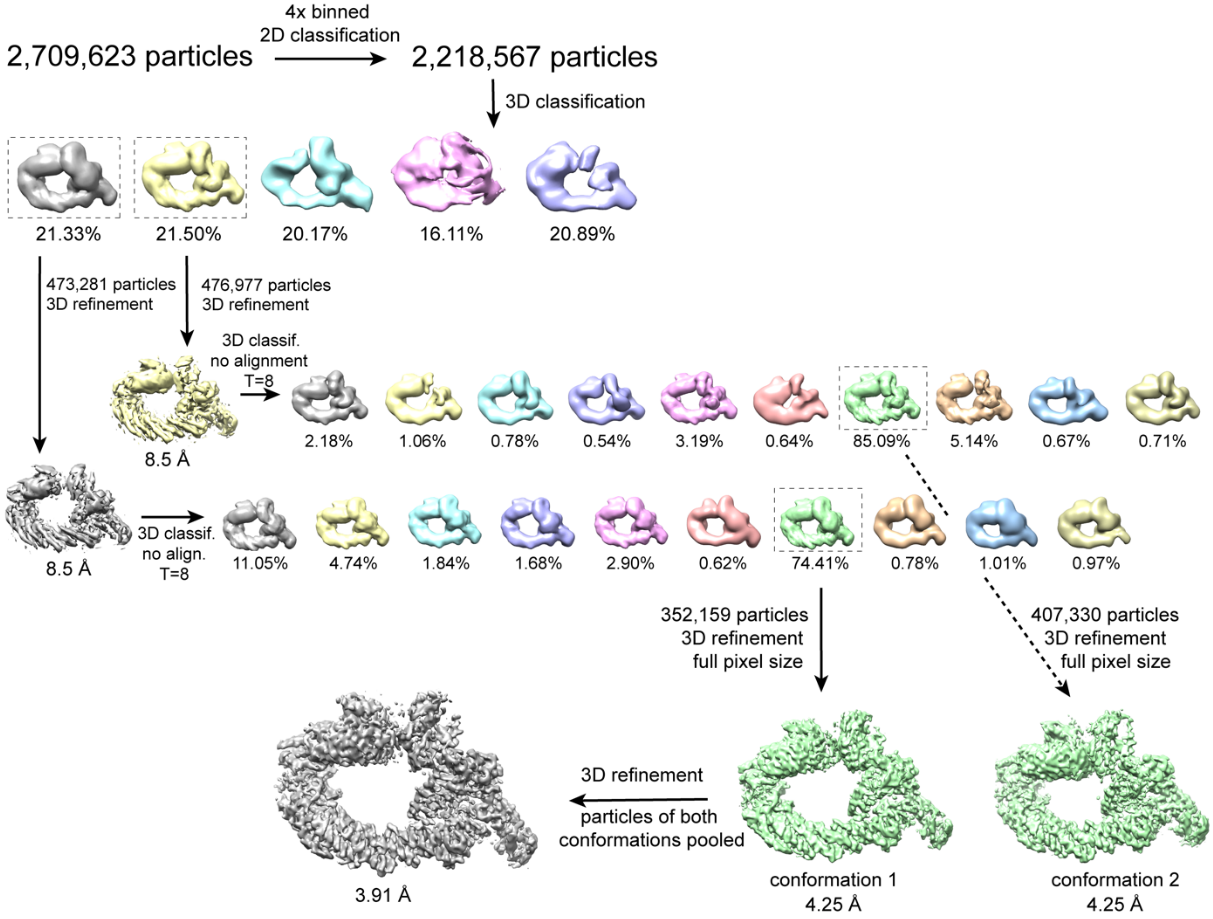

Supplemental Figure 3 | Cryo-EM image processing flowchart of the complex representing Transition State 2, SCF^{SKP2}-dependent ubiquitin transfer from ARIH1 to the substrate phospho-p27. Cryo-EM image processing flowchart. 3D classification yielded two major classes. These classes differed in their orientation of the Fbox-protein SKP2. This orientational flexibility of the Fbox protein was also seen for complexes with other substrate receptors such as FBW7 and β-TRCP (not shown). Further classifications were carried out to obtain homogenous particle sets. Both conformations were refined to an overall resolution of 4.25 Å. Pooling and collectively refining the particles of both conformations resulted in an overall increased map resolution of 3.91 Å and improved map quality of the E3-E3act domain and CUL1 scaffold.

Supplemental table 1 | Structural explanations of mutational effects on ARIH1-mediated ubiquitylation of neddylated SCF substrates

Mutation #	E3-E3 Mutation Site: Protein (Mutation)	Reference	Published Defect	Explanation Based On E3-E3 Transition State Structures
1	RBX1$^{\Delta RING}$	Scott et al., 2016	Severe (no detectable activity)	RBX1 RING is crucial component of E3/E3act superdomain
2	RBX1 (W35A)	Scott et al., 2016	Severe (no detectable activity)	RBX1 RING alignment with CUL1 in E3/E3act domain
3	RBX1 (D36P)	Scott et al., 2016	Severe (no detectable activity)	RBX1 RING alignment with CUL1 in E3/E3act domain
4	RBX1 (I37A)	Scott et al., 2016	None (WT activity)	Not in E3/E3 interface
5	RBX1 (V38P)	Scott et al., 2016	None (WT activity)	Not in E3/E3 interface
6	RBX1 (V39G)	Scott et al., 2016	None (WT activity)	Not in E3/E3 interface
7	RBX1 (D40G)	Scott et al., 2016	Mild	Linker that rotates for RING alignment with CUL1
8	RBX1 (N41G)	Scott et al., 2016	Mild	Linker that rotates for RING alignment with CUL1
9	RBX1 (N47A:H48A)	Scott et al., 2016	Mild	RBX1-Ariadne domain H-bond
10	RBX1 (D51A)	Scott et al., 2016	None (WT activity)	Not in E3/E3 interface
11	RBX1 (I54A)	Scott et al., 2016	None (WT activity)	Benign change at interface
12	RBX1 (Q57A)	Scott et al., 2016	Mild	RBX1-CUL1 domain H-bond
13	RBX1 (F81N)	Scott et al., 2016	None (WT activity)	Not in E3/E3 interface
14	RBX1 (T90A:R91A)	Scott et al., 2016	None (WT activity)	At periphery of E3/E3act domain
15	RBX1 (V93R)	Scott et al., 2016	None (WT activity)	Not in E3/E3 interface
16	RBX1 (N98Q)	Scott et al., 2016	None (WT activity)	Not in E3/E3 interface
17	RBX1 (E102V)	Scott et al., 2016	None (WT activity)	Not in E3/E3 interface
18	RBX1 (F103V)	Scott et al., 2016	None (WT activity)	Not in E3/E3 interface
19	RBX1 (Y106I)	Scott et al., 2016	None (WT activity)	Not in E3/E3 interface
20	RBX1 (H108K)	Scott et al., 2016	None (WT activity)	Not in E3/E3 interface
21	NEDD8 (Q40E)	This study	Severe (no detectable activity)	clashes with CUL1 WHB domain
22	NEDD8 (I44A)	This study	Severe (no detectable activity)	UBAL binding interface (hydrophobic)
23	Ubiquitin (I44A)	Scott et al., 2016	Severe (no detectable activity)	Ub-guided helix binding interface (hydrophobic)
24	Ubiquitin (L71A)	Scott et al., 2016	Moderate	Rcat binding interface (hydrophobic)

**25**	Ubiquitin (L73A)	Scott et al., 2016	Moderate	Rcat binding interface (hydrophobic)
**26**	Ubiquitin (R74A)	Scott et al., 2016	Mild	Rcat binding interface (salt bridge)
**27**	ARIH1 (R118A:E119A)	Scott et al., 2016	None (WT activity)	Not in E3/E3 interface
**28**	ARIH1 (V123D)	Scott et al., 2016	Severe (no detectable activity)	UBAL binding interface (hydrophobic)
**29**	ARIH1 (V123A:I124A:W140)	Kelsall et al., 2013	Severe (no detectable activity)	UBAL binding interface (hydrophobic)
**30**	ARIH1 (Q125)	Scott et al., 2016	None (WT activity)	At periphery of UBAL-NEDD8 domain
**31**	ARIH1 (I130A)	Scott et al., 2016	None (WT activity)	Not in E3/E3 interface
**32**	ARIH1 (H137A:N139A)	Scott et al., 2016	None (WT activity)	Not in E3/E3 interface
**33**	ARIH1 (E143A:K144A)	Scott et al., 2016	None (WT activity)	At periphery of UBAL-NEDD8 domain
**34**	ARIH1 (E147A)	Scott et al., 2016	None (WT activity)	At periphery of UBAL-NEDD8 domain
**35**	ARIH1 (Y149A)	Scott et al., 2016	Moderate	Positioning V123 and F150 (residues required for NEDD8 I44 binding)
**36**	ARIH1 (F150A)	Scott et al., 2016	Severe (no detectable activity)	UBAL binding interface (hydrophobic)
**37**	ARIH1 (N153A:L154A:E155A)	Scott et al., 2016	None (WT activity)	Not in E3/E3 interface
**38**	ARIH1 (K156A:L157A:F158A)	Scott et al., 2016	Severe (no detectable activity)	Stabilization of remodeled UBAL/RING1/RTI helix activated conformation
**39**	ARIH1 (E160A:C161A)	Scott et al., 2016	None (WT activity)	Not in E3/E3 interface
**40**	ARIH1 (H162A:V163A:I164A)	Scott et al., 2016	Moderate	Stabilization of remodeled UBAL/RING1/RTI helix activated conformation
**41**	ARIH1 (N165A:P166A:S166A)	Scott et al., 2016	None (WT activity)	Not in E3/E3 interface
**42**	ARIH1 (K168A:K169A:S170A)	Scott et al., 2016	None (WT activity)	Not in E3/E3 interface
**43**	ARIH1 (R171A:T172A:R173A)	Scott et al., 2016	None (WT activity)	Not in E3/E3 interface
**44**	ARIH1 (Q174A:M175A:N176A)	Scott et al., 2016	None (WT activity)	Not in E3/E3 interface
**45**	ARIH1 (T177A:R178A:S179A)	Scott et al., 2016	None (WT activity)	Not in E3/E3 interface
**46**	ARIH1 (I188A)	Scott et al., 2016	Severe (no detectable activity)	E2 binding interface (hydrophobic)
**47**	ARIH1 (Y215A)	Dove et al., 2017	Severe (no detectable activity)	E2 binding interface (hydrophobic)
**48**	ARIH1 (D241A:D242A:N243A)	Scott et al., 2016	None (WT activity)	Not in E3/E3 interface
**49**	ARIH1 (T244A:V245A:M246A)	Scott et al., 2016	None (WT activity)	Not in E3/E3 interface
**50**	ARIH1 (R247A:L248A:I249A)	Scott et al., 2016	Mild	Stabilization of remodeled UBAL/RING1/RTI helix activated conformation

**51**	ARIH1 (T250A:D251A:S252A)	Scott et al., 2016	None (WT activity)	Not in E3/E3 interface
**52**	ARIH1 (K253A:V254A:K255A)	Scott et al., 2016	Mild	Ub binding interface in remodeled UBAL/RING1/RTI helix activated conformation
**53**	ARIH1 (K257A:Y258A)	Scott et al., 2016	Severe (no detectable activity)	Central role coordinating ubiquitin and RING1
**54**	ARIH1 (K257A:H260A:L261A)	This study	Moderate	Ubiquitin binding interface
**55**	ARIH1 (Q259A:H260A)	Scott et al., 2016	None (WT activity)	At periphery of Ubiquitin binding interface
**56**	ARIH1 (L261A:I262A)	Scott et al., 2016	None (WT activity)	At periphery of Ubiquitin binding interface
**57**	ARIH1 (T263A:N264A)	Scott et al., 2016	None (WT activity)	At periphery of Ubiquitin binding interface
**58**	ARIH1 (S265A:F266A:V267A)	Scott et al., 2016	Severe (no detectable activity)	Central role coordinating IBR and UBAL in remodeled activated conformation
**59**	ARIH1 (E268A:C269A)	Scott et al., 2016	None (WT activity)	Not in E3/E3 interface
**60**	ARIH1 (N270A:R271A)	Scott et al., 2016	None (WT activity)	Not in E3/E3 interface
**61**	ARIH1 (L272A:L273A)	Scott et al., 2016	None (WT activity)	Not in E3/E3 interface
**62**	ARIH1 (K274A:W275A)	Scott et al., 2016	None (WT activity)	At periphery of Ubiquitin binding interface
**63**	ARIH1 (P279A:D280A)	Scott et al., 2016	None (WT activity)	At periphery of Ubiquitin binding interface
**64**	ARIH1 (H282A:H283A)	Scott et al., 2016	None (WT activity)	At periphery of Ubiquitin binding interface
**65**	ARIH1 (Q288A:Y289A)	Scott et al., 2016	None (WT activity)	Not in E3/E3 interface
**66**	ARIH1 (D291A:K293A)	Scott et al., 2016	None (WT activity)	Not in E3/E3 interface
**67**	ARIH1 (R296A)	Scott et al., 2016	None (WT activity)	Not in E3/E3 interface
**68**	ARIH1 (K298A)	Scott et al., 2016	None (WT activity)	Not in E3/E3 interface
**69**	ARIH1 (Q302A)	Scott et al., 2016	None (WT activity)	Not in E3/E3 interface
**70**	ARIH1 (D313A)	Scott et al., 2016	None (WT activity)	Not in E3/E3 interface
**71**	ARIH1 (W319A:L320A)	Scott et al., 2016	None (WT activity)	Not in E3/E3 interface
**72**	ARIH1 (K321A:K322A)	Scott et al., 2016	None (WT activity)	Not in E3/E3 interface
**73**	ARIH1 (W323A:I324A)	Scott et al., 2016	None (WT activity)	Not in E3/E3 interface
**74**	ARIH1 (K325A:K326A)	Scott et al., 2016	None (WT activity)	Not in E3/E3 interface
**75**	ARIH1 (C327A:D328A)	Scott et al., 2016	None (WT activity)	Not in E3/E3 interface
**76**	ARIH1 (D329A:D330A)	Scott et al., 2016	None (WT activity)	Not in E3/E3 interface
**77**	ARIH1 (S331A:E332A)	Scott et al., 2016	None (WT activity)	Not in E3/E3 interface
**78**	ARIH1 (T333A:S334A)	Scott et al., 2016	Mild	Periphery of Ubiquitin binding interface
**79**	ARIH1 (N335A:W336A)	Scott et al., 2016	Mild	Ubiquitin binding interface (hydrophobic)

**80**	ARIH1 (W336A:I337A)	This study	Severe (no detectable activity)	Ub-guided helix (hydrophobic ubiquitin binding interface)
**81**	ARIH1 (N340A:T341A)	Scott et al., 2016	Severe (no detectable activity)	Ubiquitin binding interface
**82**	ARIH1 (K342A:E343A)	Scott et al., 2016	Severe (no detectable activity)	Ubiquitin binding interface
**83**	ARIH1 (P345A:K346A)	Scott et al., 2016	None (WT activity)	Not in E3/E3 interface
**84**	ARIH1 (H348A:V349A:T350A)	Scott et al., 2016	None (WT activity)	Periphery of Ubiquitin binding interface
**85**	ARIH1 (I351A:E352A)	Scott et al., 2016	Severe (no detectable activity)	Ubiquitin binding interface
**86**	ARIH1 (C357)	Wenzel et al., 2011	Severe (no detectable activity)	catalytic cysteine
**87**	ARIH1 (N358A)	Scott et al., 2016	Mild	Catalytic Rcat conformation
**88**	ARIH1 (H359A)	Scott et al., 2016	Mild	Catalytic Rcat conformation
**89**	ARIH1 (M360A)	Scott et al., 2016	Severe (no detectable activity)	Rcat folding defect
**90**	ARIH1 (V361D)	Scott et al., 2016	None (WT activity)	Not in E3/E3 interface
**91**	ARIH1 (R363A)	Scott et al., 2016	None (WT activity)	Not in E3/E3 interface
**92**	ARIH1 (N364A:Q365A:N366A)	Scott et al., 2016	None (WT activity)	Not in E3/E3 interface
**93**	ARIH1 (K368A:E370A)	Scott et al., 2016	None (WT activity)	Not in E3/E3 interface
**94**	ARIH1 (W373A)	Scott et al., 2016	Severe (no detectable activity)	Rcat folding defect
**95**	ARIH1 (V374A)	Scott et al., 2016	None (WT activity)	Not in E3/E3 interface
**96**	ARIH1 (P378A:W379A)	Scott et al., 2016	Severe (no detectable activity)	Catalytic Rcat conformation
**97**	ARIH1 (E380A:P381A)	Scott et al., 2016	None (WT activity)	Not in E3/E3 interface
**98**	ARIH1 (G383D:S384D:A385D)	Scott et al., 2016	Mild	Catalytic Rcat conformation
**99**	ARIH1 (W386A)	Scott et al., 2016	Severe (no detectable activity)	E2 interface<br>potential catalytic role (weak density)
**100**	ARIH1 (N388A)	Scott et al., 2016	None (WT activity)	Not in E3/E3 interface
**101**	ARIH1 (N390A)	Scott et al., 2016	Hyperactive	Ariadne domain interface
**102**	ARIH1 (R391A)	Scott et al., 2016	Hyperactive	Ariadne domain interface
**103**	ARIH1 (Y392A)	Scott et al., 2016	Hyperactive	Ariadne domain interface
**104**	ARIH1 (D395A:D396A)	Scott et al., 2016	None (WT activity)	Not in E3/E3 interface
**105**	ARIH1 (A397D:K398I:A399V)	Scott et al., 2016	None (WT activity)	Not in E3/E3 interface
**106**	ARIH1 (A400N:R401Q:D402S)	Scott et al., 2016	None (WT activity)	Not in E3/E3 interface
**107**	ARIH1 (A403Q:E405A)	Scott et al., 2016	None (WT activity)	Not in E3/E3 interface
**108**	ARIH1 (D402G:A403G:	This study	Negligible	Edge of Switch helix

	Q404G)			
**109**	ARIH1 (A403G:Q404G: E405G)	This study	Negligible	Edge of Switch helix
**110**	ARIH1 (Q404G:E405G: R406G)	This study	Negligible	Edge of Switch helix
**111**	ARIH1 (E405G:R406G:S407G)	This study	Negligible	Edge of Switch helix
**112**	ARIH1 (R406G:S407G: R408G)	This study	Negligible	Edge of Switch helix
**113**	ARIH1 (Q412A)	Scott et al., 2016	None (WT activity)	Benign mutation of Switch helix
**114**	ARIH1 (Q412G:R413G: Y414G)	This study	Hyperactive and Ligation defect	Switch helix, adjacent to Ub-guided helix
**115**	ARIH1 (L415A)	Scott et al., 2016	None (WT activity)	Benign mutation of Ub-guided helix interface
**116**	ARIH1 (F416A)	Duda et al., 2013	Hyperactive	Switch helix autoinhibitory latch defect
**117**	ARIH1 (Y417A)	Scott et al., 2016	Hyperactive	Switch helix autoinhibitory latch defect
**118**	ARIH1 (N419A)	Scott et al., 2016	None (WT activity)	Not in E3/E3 interface
**119**	ARIH1 (R420A:N423A)	Duda et al., 2013	Hyperactive	autoinhibitory latch defect
**120**	ARIH1 (R420A:N423A:E503A)	Duda et al., 2013	Hyperactive	autoinhibitory latch defect
**121**	ARIH1 (H424A)	Scott et al., 2016	None (WT activity)	Not in E3/E3 interface
**122**	ARIH1 (R429A)	Scott et al., 2016	None (WT activity)	Not in E3/E3 interface
**123**	ARIH1 (F430A:E431A)	Scott et al., 2016 Duda et al., 2013	Hyperactive	Switch helix autoinhibitory latch defect
**124**	ARIH1 (F430A:E431A:E503A)	Scott et al., 2016 Duda et al., 2013	Hyperactive	Switch helix autoinhibitory latch defect
**125**	ARIH1 (H432A)	Scott et al., 2016	None (WT activity)	Not in E3/E3 interface
**126**	ARIH1 (Y435A)	Scott et al., 2016	None (WT activity)	Not in E3/E3 interface
**127**	ARIH1 (M455A:Q456A:Q457A)	Scott et al., 2016	None (WT activity)	Not in E3/E3 interface
**128**	ARIH1 (W452A)	Scott et al., 2016	Severe (no detectable activity)	E3/E3act domain -CUL1 binding
**129**	ARIH1 (K483A:K484A:N485A)	Scott et al., 2016	None (WT activity)	Not in E3/E3 interface
**130**	ARIH1 (Y476A)	Scott et al., 2016	Hyperactive	autoinhibitory latch defect
**131**	ARIH1 (N486A)	Scott et al., 2016	Mild	E3/E3act domain -RBX1 binding
**132**	ARIH1 (I489A:I490A)	Scott et al., 2016	Mild	E3/E3act domain -RBX1 binding
**133**	ARIH1 (E492A)	Scott et al., 2016	Hyperactive	autoinhibitory latch defect
**134**	ARIH1 (N493A)	Scott et al., 2016	Hyperactive	autoinhibitory latch defect
**135**	ARIH1 (Q495A)	Scott et al., 2016	Hyperactive	autoinhibitory latch defect
**136**	ARIH1 (D497A)	Scott et al., 2016	Severe (no detectable activity	E3/E3act domain -RBX1 binding
**137**	ARIH1 (E499A)	Scott et al., 2016	Hyperactive	autoinhibitory latch defect
**138**	ARIH1 (N500A)	Scott et al., 2016	None (WT activity)	Not in E3/E3 interface
**139**	ARIH1 (K522A:Q523A:K524A)	Scott et al., 2016	None (WT activity)	At periphery of E3/E3act domain
**140**	ARIH1 (Q526A)	Scott et al., 2016	Mild	At periphery of E3/E3act domain (CUL1 binding)

**141**	ARIH1 (R530A)	Scott et al., 2016	None (WT activity)	At periphery of E3/E3act domain
**142**	ARIH1 (Y531A)	Scott et al., 2016	Severe (no detectable activity	E3/E3act domain -RBX1 binding
**143**	ARIH1 (R535A)	Scott et al., 2016	Severe (no detectable activity	E3/E3act domain -RBX1 binding
**144**	ARIH1 (R537A)	Scott et al., 2016	None (WT activity)	At periphery of E3/E3act domain
**145**	ARIH1 (H452A)	Scott et al., 2016	None (WT activity)	At periphery of E3/E3act domain
**146**	ARIH1 (Y547A)	Scott et al., 2016	None (WT activity)	Not in E3/E3 interface
